# The New Anæsthetic

**Published:** 1885-01

**Authors:** Julien W. Russell

**Affiliations:** Brooklyn, N. Y.


### "THE NEW ANÆSTHETIC."

BY JULIEN W. RUSSELL, M. D. S., BROOKLYN, N. Y.

The discovery that Hydrochlorate of Cocaine when applied to sensitive surfaces has the power of producing complete insensibility to pain has certainly excited more comment than even that of chloroform. Though only discovered a few months ago, by a German medical student, it is now in the hands of almost all the medical fraternity, who are experimenting with it, so far with the most satisfactory results. The first discovery of its effects was upon the eye, but since then it has been found to give the same results when applied to any of the mucous tissues; even when injected into the ear it will produce anæsthesia; applied to wounds, it not only relieves the pain, but does not interfere with union by first intention ; in fact, it rather hastens it than otherwise. It has also been used to modify the pains of childbirth. Applied to any portion of the mucous tissue of the mouth, it shows its anæsthetic effects as fully as when applied upon the conjuctiva of the eye. By its use abscesses can be opened, and live pulps removed from the teeth with very little pain. There is a case reported in the *Medical Record* of a small tumor on the forehead which, having been first injected with the drug, was removed without causing any pain to the patient.

The formula of Hydrochlorate of Cocaine is $C_{17}$ $H_{21}$ $NO_4$ HCl. Its physiological effect, when given in large doses, is to produce tetanic convulsions, and in smaller ones, hyperæsthesia, dilation of the pupils, diminution of movement, etc. Its effects are similar to those of the alkaloids of tea, coffee, guarana and cocoa.

Dr. D. C. Cocks, Assistant Surgeon N. Y. Eye and Ear Infirmary, has just sent me a report of three cases in which he has used it. In the first two he applied the two per cent. solution to the soft palate and the walls of the pharynx. The patients had a tendency to nausea whenever a laryngoscopic mirror was introduced. Fifteen minutes after the first application the larynx was examined with the mirror without the slightest trouble. The third case was a tonsillar abscess. He painted the anterior pillar of the fauces with a four per cent. solution, and then opened the abscess, the patient complaining of very little pain.

It is very common to find patients who are so sensitive that it is almost an impossibility to either take an impression or introduce a napkin, on account of the induced nausea. In such cases cocaine, when applied in the above manner, will entirely cure this tendency, so that any operation can readily be performed. An abscess can be treated in the same way before it is opened. Apply the four per cent. solution to the place where it is about to point, the part being protected from the saliva. Five minutes after, wipe off the solution and apply some more. Leave this on for from five to ten minutes, when the incision can be made to at least four or five lines in depth.

It is in removing live pulps from the teeth that this drug plainest shows its power. Expose the pulp by removing the decay, and puncture it so that it will bleed slightly. Apply the rubber dam and dry out the cavity. After the bleeding has ceased, place a drop of the cocaine on the pulp, and repeat it every five minutes for from fifteen to twenty minutes. Upon the tissue being completely anæsthetized it can be removed in the usual manner.

Sensitive teeth that have hitherto resisted the action of all the obtunding agents in the materia medica will, in some instances at least, yield to this agent when applied in the same manner.

I do not believe that it will be of much benefit in extracting teeth, as its action will hardly penetrate through such dense tissue; but in the first stages of acute periostitis there is no doubt that, when the pain is very severe, its application at short intervals to the gum above the affected tooth will considerably allay the pain. The two per cent. solution is not strong enough to use in the mouth where the tissues are less sensitive than the eye, so it is better to use the four per cent. solution. The present cost of cocaine will for a while prevent its general adoption, but in time this will be remedied. The retail price for the four per cent. solution is about two dollars a drachm.

